# Sweet taste stimulation reduced pain ratings associated with increased blood flow in the right dorsolateral prefrontal cortex: A pilot study using NIRS

**DOI:** 10.2142/biophysico.bppb-v23.0028

**Published:** 2026-08-21

**Authors:** Yuki Okamura, Keita Sudo, Fusako Koshikawa, Etsuro Ito

**Affiliations:** 1 Department of Biology, Waseda University, Tokyo 162-0056, Japan; 2 Department of Psychology, Waseda University, Tokyo 162-8644, Japan

**Keywords:** cerebral blood flow, near-infrared spectroscopy, pain relief, dorsolateral prefrontal cortex, sweet taste

## Abstract

Oral administration of sweet stimuli to newborns is effective at alleviating pain during injections. In adults, administration of sweet stimuli prolongs the latency in perceiving experimentally induced pressure stimuli as painful. However, experimental results for adults are mixed, with some negative reports suggesting that adults do not experience analgesic effects from sweet stimuli. On the other hand, we have investigated the effects of olfactory stimulation on analgesia using near-infrared spectroscopy (NIRS), which measures changes in cerebral blood flow after olfactory stimulation. The olfactory stimulation reduced pain ratings, and a significant difference in blood flow increase in the right dorsolateral prefrontal cortex (rDLPFC) was observed when pain stimulation alone was compared with pain stimulation combined with olfactory stimulation. Furthermore, previous studies have shown that sweet stimuli increase cerebral blood flow in pain-free adults. Therefore, based on the hypothesis that sweet stimuli are involved in pain relief, the present study used NIRS to examine whether sweet stimuli affect cerebral blood flow changes targeting the rDLPFC. Pain stimulation was delivered via transcutaneous electrical stimulation. The results demonstrated that sweet taste stimulation is associated with pain relief by activating the rDLPFC, even though the score of visual analog scale used as the pain rating scale to measure the intensity of pain decreased following water stimulation. Our present study uses NIRS to measure changes in cerebral blood flow—thereby representing the intensity of pain through physiologically relevant changes—and demonstrates the effectiveness of sweet stimuli for pain relief in adults.

## Significance

Sweet taste stimulation can relieve pain in children. We investigated whether sweet stimuli also relieve pain in adults. Sweet stimuli increase cerebral blood flow in pain-free adults. Olfactory stimuli in adults have been shown to aid in pain relief, and measurement of cerebral blood flow using near-infrared spectroscopy (NIRS) has confirmed that cerebral blood flow in the right dorsolateral prefrontal cortex (rDLPFC) increases during pain relief. When adults were exposed to both sweet and painful stimuli, sweet stimuli reduced pain ratings and activated the rDLPFC. This finding supports the relationship between sweetness and pain from a neuroscientific perspective.

## Introduction

The involvement of taste stimuli in pain relief is well known from cases of oral administration of sucrose (i.e., a sweet stimulus) during the administration of injections to newborns [1,2]. This phenomenon is called sweet substance-induced analgesia (SIA) [3]. However, it has been shown that the effect of SIA weakens as human neonates or model neonatal rats grow [4,5]. Furthermore, it has been reported that sweet administration increases the latency in perceiving experimentally induced pressure stimuli as pain in adults [6], that the antinociceptive effect of sucrose is limited to those with low blood pressure [7], and that the sweet stimuli using sucrose enhance the analgesic effect of smoking [8]. On the other hand, experimental results remain mixed due to some negative reports indicating that the antinociceptive effect of sweet taste stimulation cannot be expected in adults [9].

Odor stimulation is commonly known as aromatherapy and has long been used to relieve pain [10,11]; we thus hypothesized that changes in cerebral blood flow take place by exposure to odor stimulation, leading to pain relief. To date, we have studied the effect of odor stimulation using essential oils for pain relief, suggesting that a change in cerebral blood flow could be one of the mechanisms related to the analgesic effects of aromatherapy [12]. In particular, we applied near-infrared spectroscopy (NIRS) to measure a change in cerebral blood flow upon the application of odor stimulation [13]. We used peppermint and lavender stimuli for odors, because previous studies have shown that these two odor stimuli are involved in pain relief [14,15]. We then compared pain stimulation alone and pain stimulation combined with these odor stimuli, and the results supported our hypothesis that exposure to odor stimulation produces changes in cerebral blood flow, and significant differences in blood flow in the dorsolateral prefrontal cortex (DLPFC) [12]. These results support our previous findings that the DLPFC is involved in pain relief [16]. Olfactory stimulation activates the right DLPFC (rDLPFC) [12], whereas previous direct patch stimulation at the pain site inhibited the left DLPFC (lDLPFC) [13]. One interpretation of this difference is that the contrast between rDLPFC and lDLPFC activation is important for pain relief [12].

NIRS is a useful method for measuring changes in brain activity and is highly regarded for its non-invasive nature. NIRS can measure changes in the concentration of oxygenated hemoglobin and deoxygenated hemoglobin near the brain surface, i.e., changes in cerebral blood flow, in real time [17]. Moreover, it can overcome various problems that other brain imaging techniques have, such as the participant’s posture, spatial and temporal resolution, loud noise during measurement, and the need for a shielded room. When neural activity is activated in a specific area of the brain, the local blood volume increases at that location. By measuring this change in blood flow with near-infrared light, the activated area can be explored.

Near-infrared light (especially at wavelengths of 700 to 900 nm) has the property of easily penetrating skin and bones and has different optical absorption characteristics for oxygenated hemoglobin (oxyHb) and deoxygenated hemoglobin (deoxyHb) in the blood. When near-infrared light is irradiated through the scalp, it diffuses to a depth of about 30 mm, and the diffusely reflected light can then be measured at a site about 30 mm away. The measured light contains relative information on the amount of oxyHb, deoxyHb, and total hemoglobin. Moreover, oxygen consumption changes that coincide with neural activity in the brain are reflected as a change in blood flow. Immediately after neural activity begins, the amount of oxyHb increases, and the amount of deoxyHb decreases. When activity ends, both return to base levels. These types of changes can actually be quantified as changes in the light absorption waveform detected by the detection sensor.

Previous NIRS studies have shown that sweet stimuli increased the cerebral blood flow in the DLPFC [18]. Consequently, it is necessary to investigate whether this blood increase is associated with pain relief. In the present study, working under the assumption that sweet stimuli are involved in pain relief, we used NIRS to investigate whether they affect changes in cerebral blood flow. As a pain stimulus, electrical stimulation was administered. The results suggest that sweet taste stimuli contribute to pain relief by activating the rDLPFC.

## Materials and methods

### Participants

The present study was carried out in accordance with the recommendations of the principles and guidelines of the Declaration of Helsinki. All participants provided written informed consent to participate after they were informed of the safety of NIRS and the purpose of the experiments. The protocol was approved by the Office of Research Ethics (ethics review concerning research with human subjects) of Waseda University (2024-035, April 25, 2024). Data from 18 volunteer participants (right-hand-dominant, ages 20–25 years) were available for analysis, including 14 men and 4 women. The participants were from the Tokyo area in Japan, self-reported experiencing no pain, were non-smokers, and had to abstain from food for one hour before the experiments.

### Experimental protocol

The experimental schedule is as shown in Figure 1. The pain-only stimulation experiment was performed first (Figure 1A). Three min later, the pain and sweetness stimulation experiment was performed (Figure 1B). Finally, 3 min later, pain-only stimulation was performed with a weaker current. See the next section for the strength of the electrical stimulation. Furthermore, the sweet stimuli and water stimuli experiments were conducted on different days to avoid the influence of habituation.

### Pain stimulation and pain measurement

Transcutaneous electrical stimulation (TCES, Pain Vision, PS 2100; NIPRO, Osaka, Japan) was used for pain stimulation because the ‘degree of pain’ can be calculated from the current perception threshold, which was defined as the lowest electrical current detected by each participant [13]. TCES stimulation (pulsed current with bipolar electrode placement) was performed near the cubital fossa on the right forearm. The minimum sensory threshold was measured for each participant, and a current with a pain level of 800 was applied as the pain stimulus. The degree of pain was calculated using the following formula: [(pain-equivalent current–minimum perceived current)/minimum perceived current]×100. The minimum perceptible current is the smallest current that the participant can sense, and is measured before the experiment. This normalization based on the minimum perceived current corrects for individual differences in sensory thresholds between participants and standardizes the intensity of pain stimuli. The pain-inducing current is the strength (mA) of the current that actually flows. By setting the pain level at 800, it is possible to pass a current that causes the same level of pain to each participant. The current was set to 800 as this placed it within a range that enabled participants to feel pain while still being safe. A visual analog scale (VAS) was used as the pain rating scale to measure the intensity of pain (integer scale: 0–10; in increments of 0.1). The amount of pain experienced by a participant falls along a continuum from none to an extreme amount of pain. From the participant’s perspective, this spectrum appears continuous, as the categorizations of none, mild, moderate, and severe would suggest. The VAS used in the present study was a straight horizontal line of fixed length (100 mm). Finally, a weak electric current of TCES was applied to evaluate the VAS and the NIRS responses (Pain level=500). The purpose of this weak electric current experiment was to investigate how the weakening of the pain stimulus itself changes the brain activity involved in pain processing.

### Taste stimulation

An aqueous solution of sucrose was used for sweetness. Previous studies have used sucrose at a concentration of 24% w/v, so we followed suit and decided to use 7.2 g in 30 mL [9]. The participants were asked to start drinking as soon as the electric current was applied and to keep neck movements to a minimum while drinking. No information regarding conditions such as taste or the duration of the experiment was disclosed to participants.

### NIRS measurements

NIRS (ETG-4000/OT-R40; Fujifilm Healthcare Systems Corporation, Tokyo, Japan) was used to measure the relative concentration of oxyHb flow over the prefrontal cortex, including the DLPFC and orbitofrontal cortex (OFC). NIRS experiments were conducted in a quiet room at a room temperature of 22–23°C. Participants wore earphones and were exposed to environmental sounds (the sound of a babbling river and chirping birds) to promote relaxation and psychological equilibrium. Participants were given instructions to rest, close their eyes, and remain awake.

Details of NIRS measurements have been described previously [13]. Briefly, the NIRS optodes were placed in the proper position for each participant according to the international 10–20 system [19]. According to the atlas by Talairach and Tournoux [20], the dorsomedial prefrontal cortex (DMPFC) was covered by Ch. 2, 3, 7, 11, and 12; the ventromedial prefrontal cortex (VMPFC) was covered by Ch. 16; the lDLPFC was covered by Ch. 4, 8, 9, 13, 17, and 18; the rDLPFC was covered by Ch. 1, 5, 6, 10, 14, and 15; the left OFC (lOFC) was covered by Ch. 21 and 22; and the right OFC (rOFC) was covered by Ch. 19 and 20 (Figure 2). The ventrolateral prefrontal cortex (VLPFC) was not measured. Absorption of NIR light is caused by oxyHb and deoxyHb. Although they have different absorption spectra, the isosbestic point is around 805 nm. Therefore, it is possible to calculate the change in concentration of oxyHb and deoxyHb by measuring the absorbance change at two wavelengths (780 nm and 830 nm), taking into account the molar molecular extinction coefficients of oxyHb and deoxyHb. We analyzed oxyHb in our experiments to calculate the area under the curve (AUC). The NIRS data were analyzed with R (version 4.2.2; https://www.r-project.org/). Participants were asked to remain at rest starting one min prior to the measurement, and a baseline was established by setting the average value from 5 sec before the start of measurement to the start time itself as zero. The sampling rate of the NIRS was 10 Hz. To reduce motion artifacts associated with drinking, swallowing, heartbeat and body movements, a 0.01–1 Hz finite impulse response band-pass filter and a moving average process were applied to the raw data. The NIRS data were averaged using a moving average, and the values of the 3 trials were averaged.

### Statistics

A *p*-value of 0.05 was considered significant. For the VAS values and NIRS data, a Wilcoxon signed-rank test was used. When 22 NIRS channels were measured, the *p*-values were corrected by a multiple comparison of false discovery rate in the Benjamini–Hochberg method. The data of Cohen’s *d* were shown as the effect size. The statistical calculations were analyzed with R (version 4.2.2; https://www.r-project.org/).

## Results

### Sweet stimulus-induced pain relief (VAS measurements)

Pain relief was measured using a VAS when pain relief treatment using sweet stimuli was used (Figure 3). A Wilcoxon signed-rank test showed a significant difference (*p*<0.001, Cohen’s *d*=0.714, N=18), and only one of the 18 participants did not experience any reduction in pain. Here, we refer to this participant who did not feel pain relief from sweet stimuli as Participant A.

### Sweet stimulus-induced NIRS oxyhemoglobin changes

We compared the oxyHb changes observed with NIRS between “pain only” and “pain+sweetness”. Cerebral blood flow was significantly increased in Ch. 1 (Figure 4A) and Ch. 6 (Figure 4B), belonging to the rDLPFC regions (Figure 4C). For this channel, the AUC change was obtained as *p*=0.030, Cohen’s *d*=0.708 for Ch. 1 (N=18) and *p*=0.043, Cohen’s *d*=0.530 for Ch. 6 (N=18). The cerebral blood flow of Participant A, shown in the previous section, increased in Ch. 1, but decreased in Ch. 6, when this person was given a sweet stimulus. Two participants experienced decreased blood flow in only Ch. 1, four participants experienced decreased blood flow in only Ch. 6, and two participants experienced decreased blood flow in both Ch. 1 and Ch. 6.

### Water stimulus (placebo)-induced pain relief (VAS measurements)

Pain relief was measured using the VAS when pain relief treatment using water stimuli, i.e., a placebo, was administered (Figure 5). A Wilcoxon signed-rank test showed a significant difference (*p*<0.001, Cohen’s *d*=0.357, N=18).

### Water stimulus (placebo)-induced NIRS oxyhemoglobin changes

When pain relief treatment was presented using water stimulation, changes in the oxyHb concentration were measured using NIRS, and a Wilcoxon signed-rank test was performed to compare “pain only” and “pain+water”. Cerebral blood flow significantly increased in Ch. 1 (Figure 6A). In contrast, no significant change was observed in Ch. 6 (Figure 6B). These channels belong to the rDLPFC region. The absence of a significant Ch. 6 response under water stimulation (*p*=0.932) may be important evidence for sweetness-specific rDLPFC activation. No significant differences were observed in any other channels. Therefore, only Ch. 1 showed a significant increase in cerebral blood flow under water stimulation. In the case of Participant A, water stimulation increased cerebral blood flow in Ch. 1 but decreased it in Ch. 6.

### Sweet stimulus reduces pain ratings more than water stimulus

We showed the comparison of VAS change scores between the sweet stimulus and water stimulus conditions. We used the Wilcoxon signed-rank test to compare these two values: the difference between the VAS score for pain alone and the VAS score when sweetness and pain were administered, and the difference between the VAS score for pain alone and the VAS score when water and pain were administered. The results showed *p*<0.001 and Cohen’s *d*=0.78, indicating that the administration of sweetness resulted in a greater perceived reduction in pain (Figure 7).

### Comparison of NIRS oxyhemoglobin changes between “pain+sweetness” and “pain+water” stimuli

When pain relief treatment was administered using a sweet stimulus, a change in the oxyHb concentration was measured using NIRS, and a Wilcoxon signed-rank test was performed to compare the changes in oxy-Hb AUC between the sweet stimulus and water stimulus; the results showed *p*=0.545, Cohen’s *d*=0.379 for Ch. 1 and *p*=0.520, Cohen’s *d*=0.504 for Ch. 6, indicating no significant difference (Figure 8).

### Responses to the lower pain level

At the end of the experiments, a weak electric current of TCES was applied to evaluate the VAS and the NIRS responses (Pain level=500). In this case, no sweet stimulus was given, only a weak electric current. Of the 18 participants, two did not notice this weak electrical stimulation, but 16 did. The two who did not notice the stimulation are referred to as Participant B and Participant C. Figure 9A shows the NIRS data for Participant B, Figure 9B shows that for Participant C, and Figure 9C shows that for the one typical participant who did notice the reduced pain level. Both Participant B and the participant who noticed the reduced current experienced a decrease in cerebral blood flow (Figure 9A, C). However, Participant C showed an unusual change in NIRS response (Figure 9B): their VAS score was 6 the first time, 6 the second time, and 7 the third time, for an average of 6.333. In addition, their VAS score was 7 when the current was weakened.

## Discussion

Previous studies have shown that sweet stimuli promote pain relief in newborns [1,2]. However, it is unclear whether the same holds true for adults [9]. In the present study, we investigated whether sweet stimuli contribute to pain relief by recording changes in cerebral blood flow in the rDLPFC using NIRS. The rDLPFC plays an important role in pain relief [16], and our results demonstrate that sweet stimuli are involved in pain relief in adults.

The DLPFC is thought to carry out advanced processes unique to humans, such as recognizing pain (determining its significance), deciding on pain-related behaviors (how to behave, how to express it to others, etc.), and recognizing social significance (how one will be perceived by others when one reports pain) [21]. The cognitive functions of the DLPFC can also work to suppress pain [22]. It is thought that pain is suppressed by unconsciously or consciously determining the biological and social significance of pain. From a pain science perspective, it is known that a descending inhibitory function operates from the DLPFC to the midbrain central gray matter.

Although Ch. 5, 10, 14, and 15 are sometimes defined as the rDLPFC, it is possible that these channels cover areas other than the rDLPFC. It is also possible that the response in the present study was localized rather than global. Sweet stimuli reduced pain ratings via Ch. 1 and 6, whereas olfactory stimuli are involved in pain relief via Ch. 15 [12]. Furthermore, considering the data in Figures 6 and 8 of the present study, our results suggest that while an increase in cerebral blood flow was observed in Ch. 1 in response to both sweet tastes and water, an increase was observed in Ch. 6 only in response to sweet stimuli; therefore, Ch. 6 is deeply involved in pain relief mediated by sweetness.

No significant difference was found in the change in oxyHb concentration in Figure 8, when pain relief treatment was administered using a sweet stimulus. One possible reason for the lack of a significant difference is that NIRS measures relative changes—setting the start of measurement as zero—rather than absolute values. Because the experiments for the sweet stimulus and water stimulus were conducted on different days, slight variations in baseline conditions may have caused a shift in the reference point for relative evaluation, potentially resulting in the absence of a detectable difference between the two conditions.

In the present study, we found that sweet stimuli reduce pain ratings. However, some may criticize this result as being due to habituation caused by repeated painful stimuli. In our experiments, we maintained an interval of at least 3 min between measurements, which we believe minimizes the influence of habituation. Furthermore, the sweet stimuli and water stimuli experiments were conducted on different days, which also suggests that there is no influence of habituation.

The purpose of the weak current experiments, as shown in Figure 7, was to investigate how brain activity involved in pain processing changes as the pain stimulus itself weakens. The results suggest that the brain activity involved in pain processing may have decreased overall. In other words, when the physical intensity of pain decreases, the entire brain, including the prefrontal cortex, is not activated, which may result in a decrease in cerebral blood flow. We consider Participant C to be exceptional. He/She likely possesses a high tolerance for pain, though, to be honest, we cannot analyze that aspect in depth at this stage and thus cannot say for certain.

When the current of the pain stimulus was reduced at the end of the experiments, many participants (16 out of 18) noticed a reduction in pain. NIRS data from the sweet taste stimulation showed a decrease in cerebral blood flow in the rDLPFC. These results show that cerebral blood flow is lower when the current is weakened compared to when a current of normal strength is flowing. This result contradicts the hypothesis that cerebral blood flow increases when pain is relieved. In turn, this suggests that the weakening of the pain itself may result in an overall decrease in brain activity involved in pain processing. In other words, when the physical intensity of pain decreases, the entire brain, including the prefrontal cortex, is less activated, resulting in a decrease in cerebral blood flow.

We found the phenomenon that the OxyHb signal in Figure 4D drops slightly 10 sec before the electrical stimulation begins. Upon reviewing many of the raw data, this drop was observed in almost all of them. Investigating the cause, it was discovered that the Pain Vision electrical stimulation device emits a faint light on its screen 10 sec before stimulation, and the participants’ attention is slightly drawn to this light. This is believed to be the reason for the drop in OxyHb.

Various reports have already been published on the effect of taste on pain relief [23]. However, to our knowledge, our present study is the first to use NIRS to measure changes in cerebral blood flow, objectively indicate the degree of pain, and demonstrate that sweet stimuli are effective in alleviating pain in adults.

## Conclusion

Sweet taste stimuli appear to be useful for pain relief. Because the concepts of pain and pain relief are highly subjective, we sought to bring objectivity to these concepts and solidify our interpretation by measuring changes in cerebral blood flow using NIRS. We believe that an increase in blood flow in the rDLPFC with a sweet stimulation, or, as shown in our previous paper, the contrast between the activation of the rDLPFC and lDLPFC, appears to be important.

## Conflict of interest

E.I. was employed by the company BioPhenoMA Inc. The other authors declare no conflicts of interest. The funder had no role in the design of the study; in the collection, analyses, or interpretation of data; in the writing of the manuscript; or in the decision to publish the results.

## Author contributions

Conceptualization, E.I.; methodology, Y.O.; software, Y.O. and K.S.; investigation, Y.O. and K.S.; data curation, Y.O. and K.S.; writing—original draft preparation, Y.O. and E.I.; writing—review and editing, K.S. and F.K.; funding acquisition, F.K. All authors have read and agreed to the published version of the manuscript.

## Data availability

The evidence data generated and/or analyzed during the current study are available from the corresponding author on reasonable request.

## Figures and Tables

**Figure 1:**
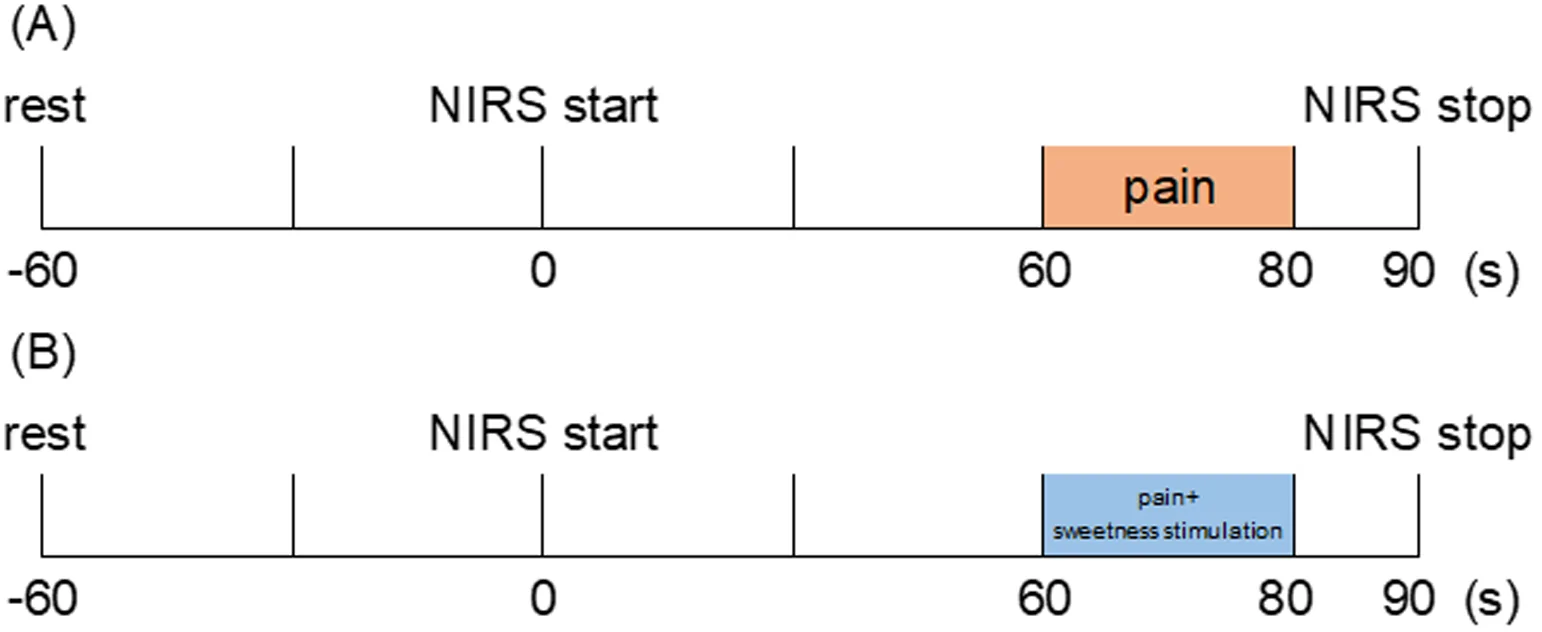
Time flow of pain and sweet stimulation experiments. (A) Experimental procedure in which only the pain stimulus is presented. Participants were seated in a chair and resting, and 60 s after starting NIRS measurements, a pain stimulus (TCES current) was applied for 20 s. See below for the details of TCES. The change in cerebral blood flow (the mean value of the AUC of the NIRS oxyHb data) was determined. See below for the details of AUC. (B) Experimental procedure in which the simultaneous stimulation of pain and sweet taste was presented. In this experiment, data on water stimulation as a placebo instead of sweet stimulation were also measured on a separate day.

**Figure 2:**
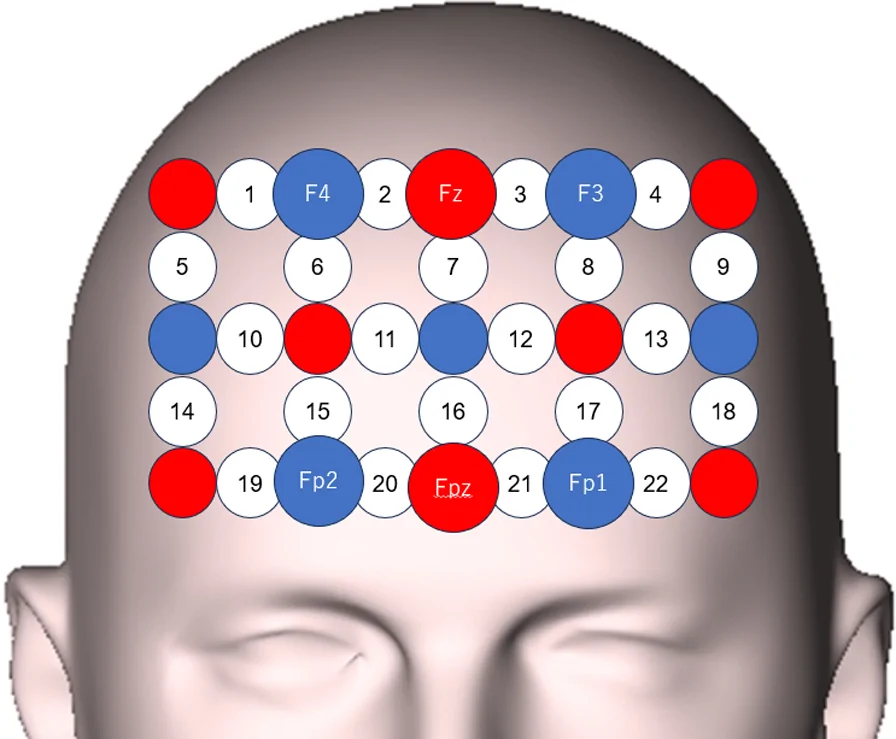
Picture of NIRS optodes. The NIRS comprised 8 elements for the NIR light emitter (red) and 7 elements for the detector (blue). The numbers indicate the channels (white) recorded. The NIRS channels were placed above the dorsolateral prefrontal cortex and the orbitofrontal cortex. The channels between the optodes measured on the skull were assigned to the brain regions using the virtual registration method. Based on the international 10–20 system, the measurement probe was positioned near F4, corresponding to the rDLPFC. Accordingly, Ch. 6 was interpreted as reflecting activity in the surrounding region of the rDLPFC. Fz is located at the frontal midline and is interpreted to reflect activity in the medial prefrontal cortex, including the DMPFC. F3 is positioned over the left frontal region and is considered to correspond to activity in the lDLPFC. Fp1 and Fp2 are located in the frontopolar region and are interpreted to reflect activity in the bilateral frontopolar cortex, as well as surrounding regions of the VMPFC and the OFC. Fpz is positioned at the frontopolar midline and is considered to reflect activity in the medial prefrontal cortex, including the frontopolar cortex and the VMPFC.

**Figure 3:**
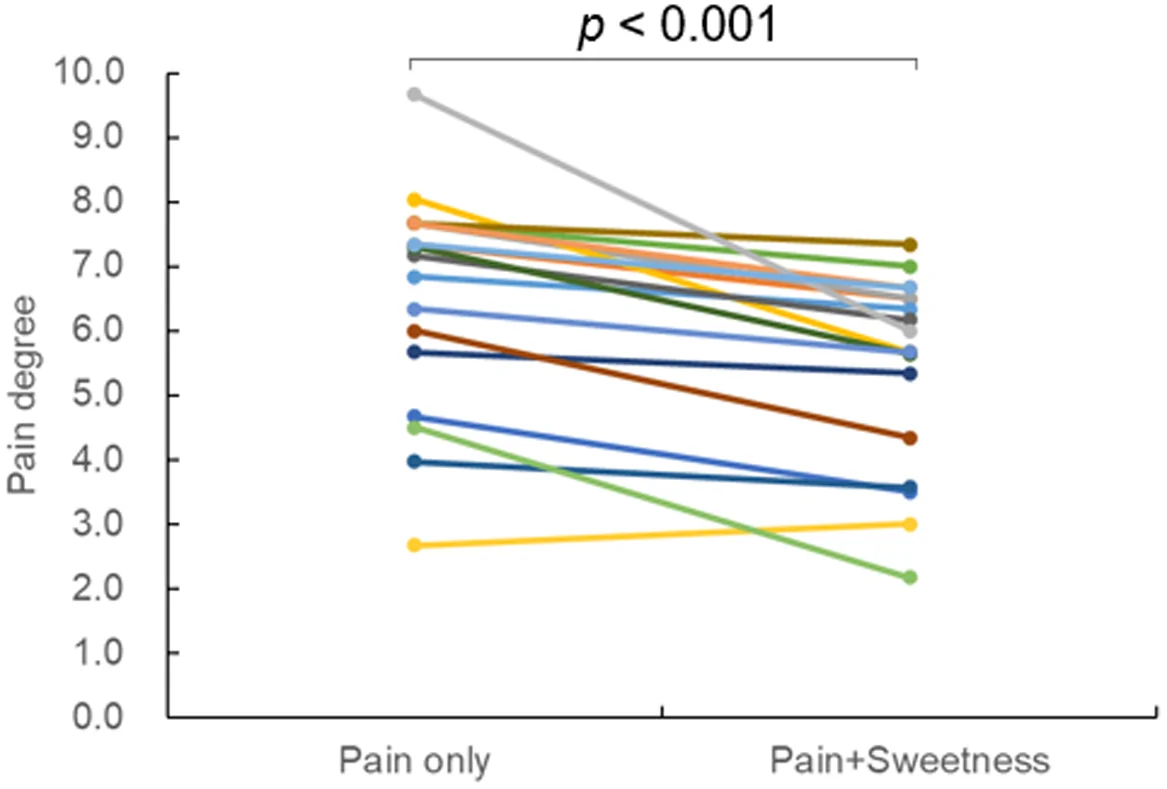
Changes in VAS values (degree of pain) by sweet taste stimulation. *p*<0.001, Cohen’s *d*=0.714, N=18. Only one of the 18 participants did not experience any reduction in pain.

**Figure 4:**
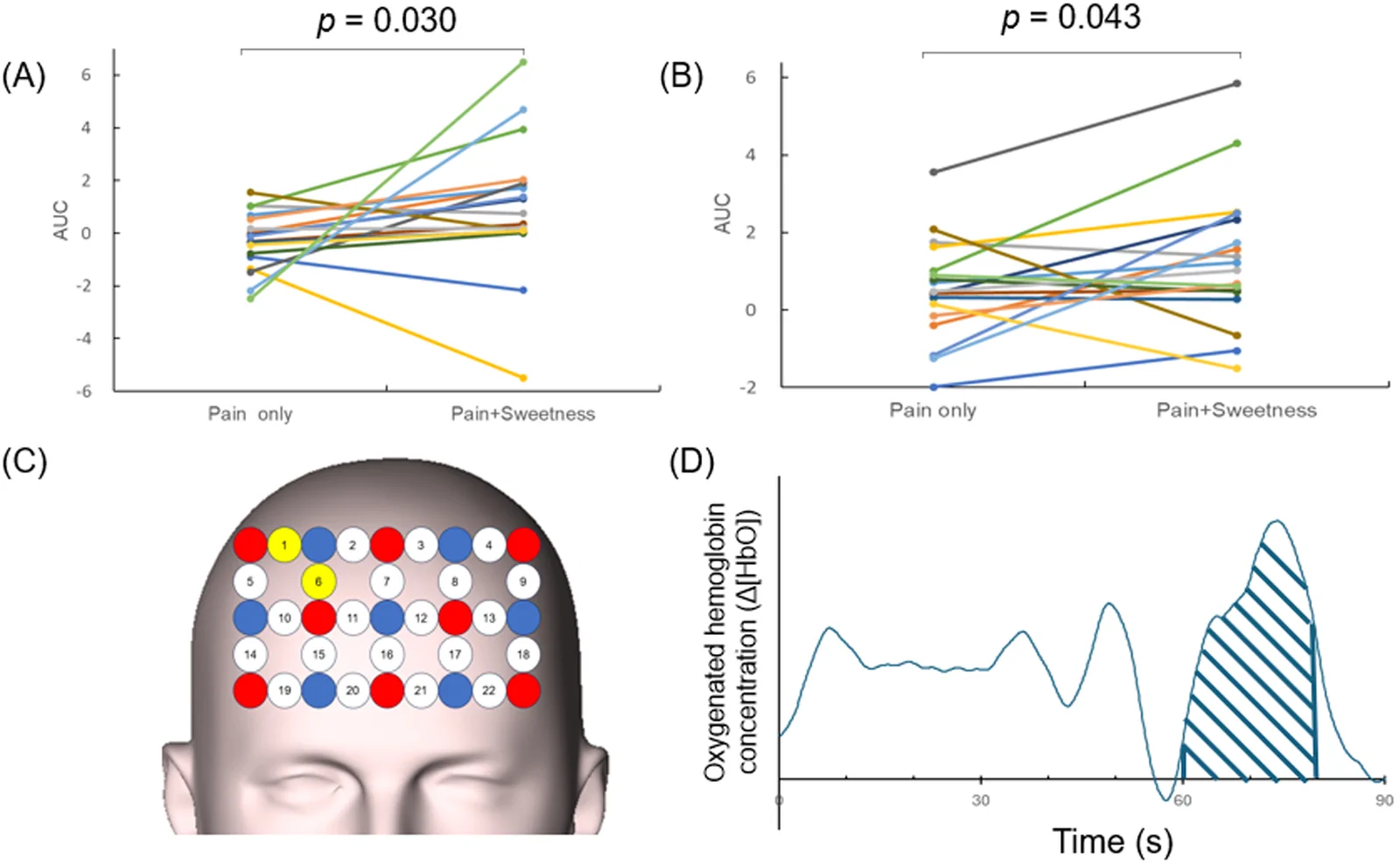
Changes in cerebral blood flow by both pain stimulation and sweet taste stimulation. (A) Ch. 1. Sweet taste stimulation increased blood flow (*p*=0.030, Cohen’s *d*=0.708, N=18). (B) Ch. 6. *p*=0.043, Cohen’s *d*=0.530, N=18. (C) NIRS channels. Yellow circles indicate the channels (i.e., rDLPFC) showing the changes in cerebral blood flow. (D) A typical graph of the AUC to analyze oxyHb. The AUC was calculated based on cerebral blood flow for 20 sec starting 60 sec after the onset of measurement.

**Figure 5:**
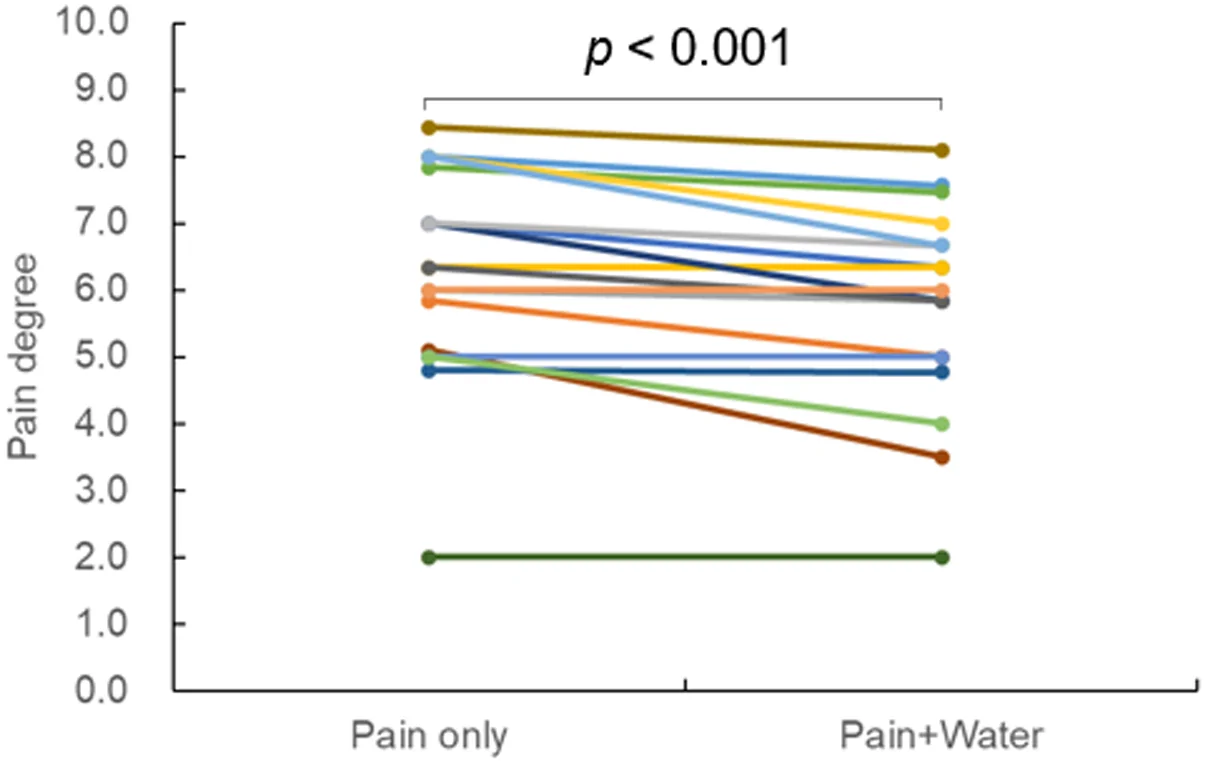
Changes in VAS values (degree of pain) by water stimulation (placebo). *p*<0.001, Cohen’s *d*=0.357, N=18. Fourteen of the 18 participants experienced some reduction in pain, even with the application of water stimulation.

**Figure 6:**
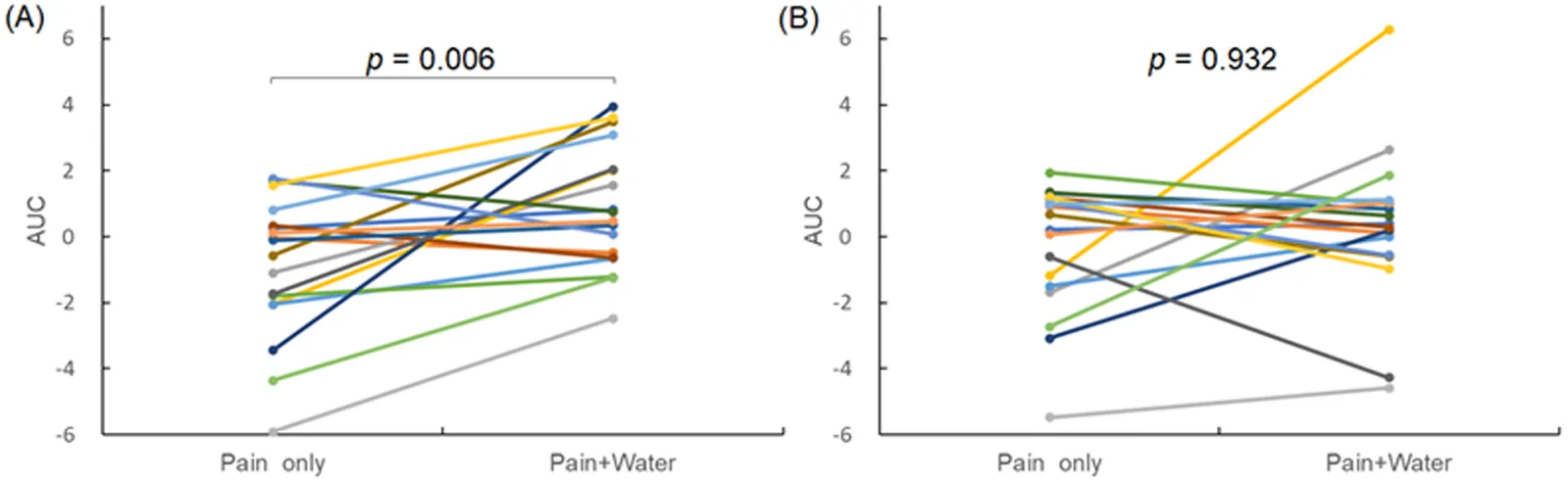
Changes in cerebral blood flow by both pain stimulation and water stimulation (placebo). (A) Ch. 1. Water stimulation increased blood flow (*p*=0.006, Cohen’s *d*=0.919, N=18). (B) Ch. 6. (*p*=0.932, Cohen’s *d*=0.285, N=18.) That is, only the data obtained in Ch. 6, shown in Figure 4B, should be noted.

**Figure 7:**
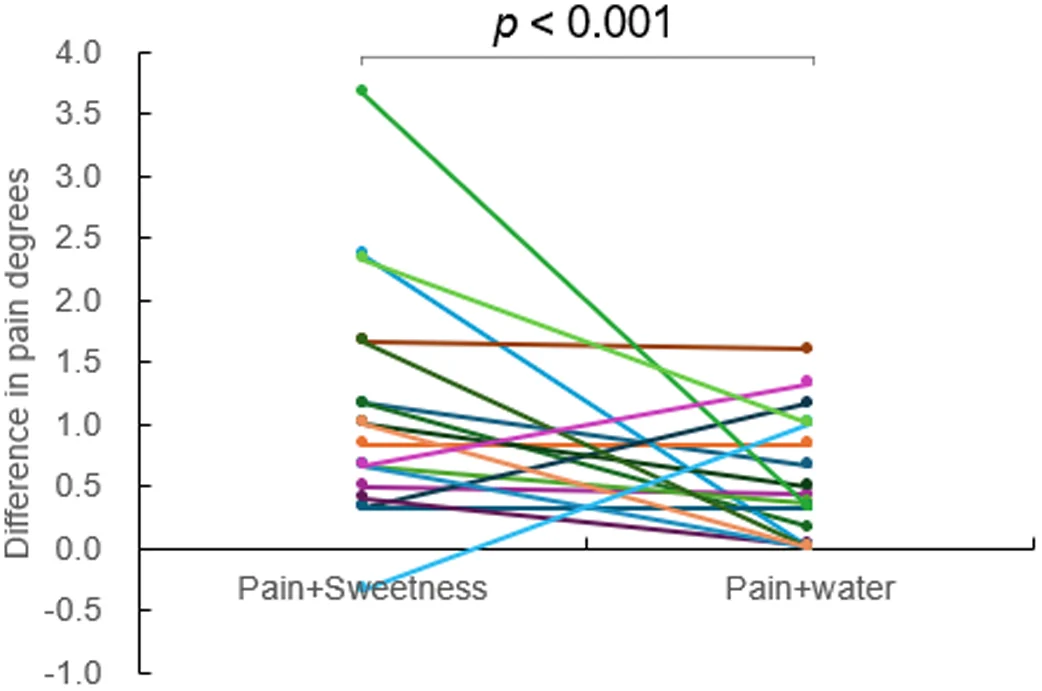
Difference in VAS values (degree of pain) between “pain+sweetness” and “pain+water” (*p*<0.001, Cohen’s *d*=0.78, N=18).

**Figure 8:**
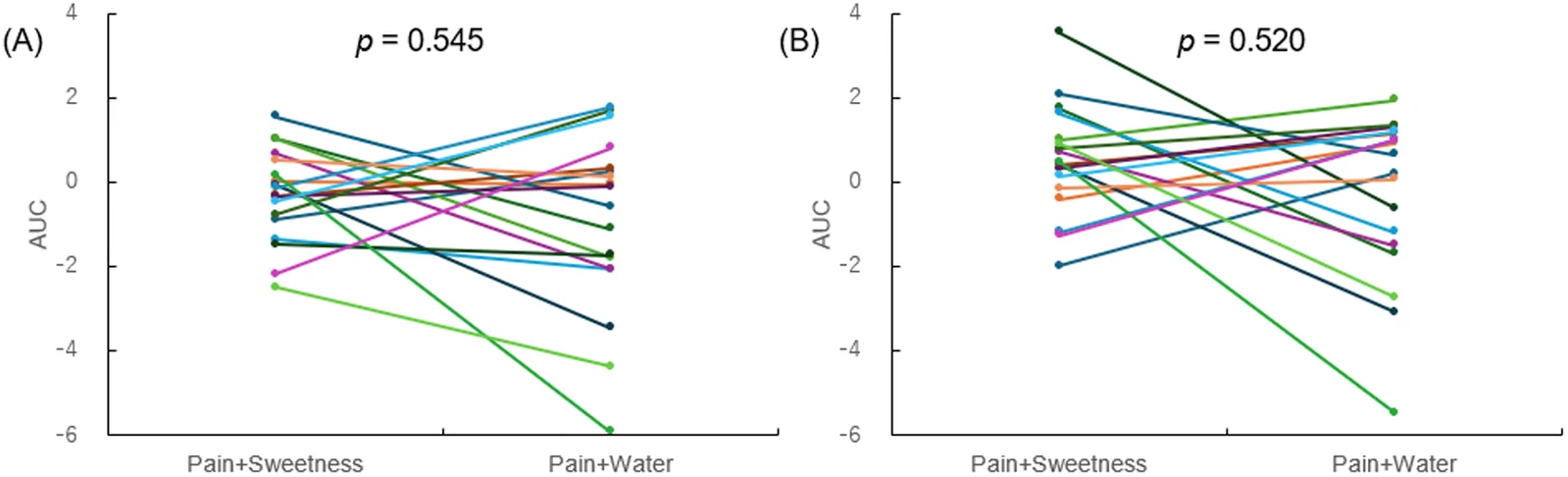
No significant differences in cerebral blood flow by both “pain+sweetness” and “pain+water”. (A) Ch. 1. (*p*=0.545, Cohen’s *d*=0.379, N=18). (B) Ch. 6. (*p*=0.520, Cohen’s *d*=0.504, N=18.)

**Figure 9:**
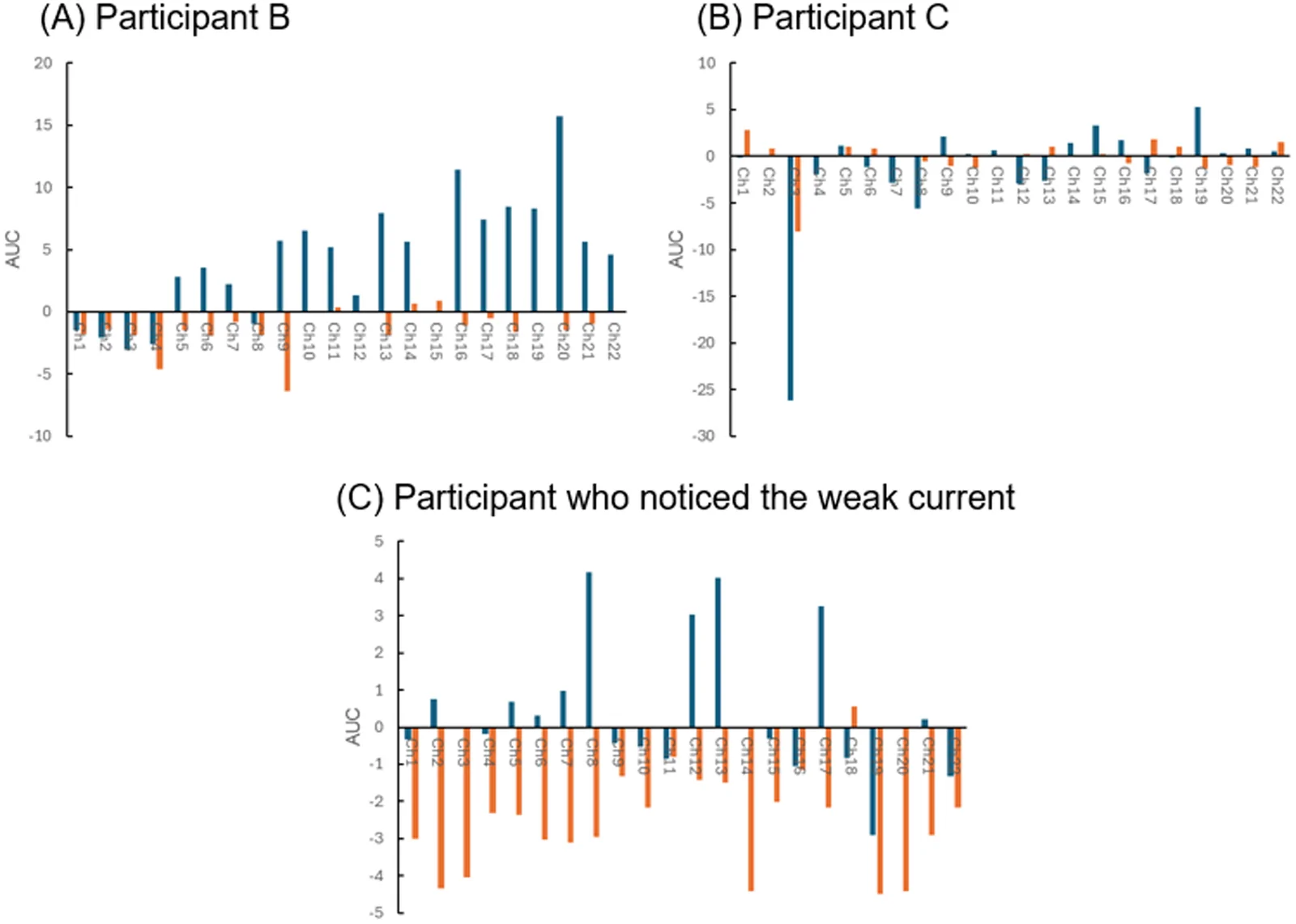
Changes in cerebral blood flow by strong TCES stimulation (a pain level of 800, shown as blue bars) and weak TCES stimulation (a pain level of 500, shown as orange bars). (A) NIRS data for Participant B. (B) NIRS data for Participant C. (C) NIRS data for a typical participant who noticed the weak current (a pain level of 500).

## References

[1] Gibbins, S., Stevens, B. Mechanisms of sucrose and non-nutritive sucking in procedural pain management in infants. Pain Res. Manag. 6, 21–28 (2001). https://doi.org/10.1155/2001/376819 10.1155/2001/376819 11854758

[2] Liu, Y., Huang, X., Luo, B., Peng, W. Effects of combined oral sucrose and nonnutritive sucking (NNS) on procedural pain of NICU newborns, 2001 to 2016: A PRISMA-compliant systematic review and meta-analysis. Medicine 96, e6108 (2017). https://doi.org/10.1097/MD.0000000000006108 10.1097/MD.0000000000006108 PMC5313029 28178172

[3] Irusta, A. E., Savoldi, M., Kishi, R., Resende, G. C., Freitas, R. L., Carvalho, A. D., et al. Psychopharmacological evidences for the involvement of muscarinic and nicotinic cholinergic receptors on sweet substance-induced analgesia in *Rattus norvegicus*. Neurosci. Lett. 305, 115–118 (2001). https://doi.org/10.1016/s0304-3940(01)01824-9 10.1016/s0304-3940(01)01824-9 11376897

[4] Barr, R. G., Quek, V. S., Cousineau, D., Oberlander, T. F., Brian, J. A., Young, S. N. Effects of intra-oral sucrose on crying, mouthing and hand-mouth contact in newborn and six-week-old infants. Dev. Med. Child. Neurol. 36, 608–618 (1994). https://doi.org/10.1111/j.1469-8749.1994.tb11898.x 10.1111/j.1469-8749.1994.tb11898.x 8034123

[5] Anseloni, V. C., Weng, H. R., Terayama, R., Letizia, D., Davis, B. J., Ren, K., et al. Age-dependency of analgesia elicited by intraoral sucrose in acute and persistent pain models. Pain 97, 93–103 (2002). https://doi.org/10.1016/s0304-3959(02)00010-6 10.1016/s0304-3959(02)00010-6 12031783

[6] Mercer, M. E., Holder, M. D. Antinociceptive effects of palatable sweet ingesta on human responsivity to pressure pain. Physiol. Behav. 61, 311–318 (1997). https://doi.org/10.1016/s0031-9384(96)00400-3 10.1016/s0031-9384(96)00400-3 9035263

[7] Lewkowski, M. D., Ditto, B., Roussos, M., Young, S. N. Sweet taste and blood pressure-related analgesia. Pain 106, 181–186 (2003). https://doi.org/10.1016/s0304-3959(03)00333-6 10.1016/s0304-3959(03)00333-6 14581126

[8] Kanarek, R. B., Carrington, C. Sucrose consumption enhances the analgesic effects of cigarette smoking in male and female smokers. Psychopharmacology 173, 57–63 (2004). https://doi.org/10.1007/s00213-003-1699-0 10.1007/s00213-003-1699-0 14722703

[9] Pepino, Y. M., Mennella, J. A. Sucrose-induced analgesia is related to sweet preferences in children but not adults. Pain 119, 210–218 (2005). https://doi.org/10.1016/j.pain.2005.09.029 10.1016/j.pain.2005.09.029 PMC1364537 16298489

[10] Lee, H. W., Ang, L., Kim, J. T., Lee, M. S. Aromatherapy for symptom relief in patients with burn: A systematic review and meta-analysis. Medicina 58, 1 (2021). https://doi.org/10.3390/medicina58010001 10.3390/medicina58010001 PMC8781071 35056310

[11] Izadi, F., Mazhari, S. A., Najafi, M., Ashoobi, M. T., Sarafi, M., Karkhah, S., et al. The effect of aromatherapy with lavender on pain of needle insertion and severity of restless legs syndrome in hemodialysis patients; a systematic review and meta-analysis. Arch. Acad. Emerg. Med. 12, e4 (2023). https://doi.org/10.22037/aaem.v12i1.2071 10.22037/aaem.v12i1.2071 PMC10674070 38022715

[12] Okamura, Y., Takayama, S., Namiki, K., Koshikawa, F., Ito, E. Near-infrared spectroscopic study of blood flow changes in the dorsolateral prefrontal cortex during pain relief by odor stimulation. Biophys. Physicobiol. 22, e220001 (2024). https://doi.org/10.2142/biophysico.bppb-v22.0001 10.2142/biophysico.bppb-v22.0001 PMC11876804 40046556

[13] Miyashiro, S., Yamada, Y., Nagaoka, M., Shima, R., Muta, T., Ishikawa, H., et al. Pain relief associated with decreased oxyhemoglobin level in left dorsolateral prefrontal cortex. PLoS One 16, e0256626 (2021). https://doi.org/10.1371/journal.pone.0256626 10.1371/journal.pone.0256626 PMC8382195 34424921

[14] Shinno, A., Isawa, M., Itoh, H., Kose, N., Ishino, H., Nishimura, T., et al. Effectiveness of aromatherapy evaluated with subjective and objective cognitive indicators: A clinical trial on young adults using near-infrared spectroscopy. Jpn. J. Pharm. Health Care Sci. 38, 265–271 (2012). https://doi.org/10.5649/jjphcs.38.265

[15] Kouzuma, N., Yamakawa, T. Associations between autonomic nervous system activity, cerebral blood flow, and essential oil preferences across the menstrual cycle. Integr. Med. Rep. 3, 9–18 (2024). https://doi.org/10.1089/imr.2023.0032

[16] Ito, E., Oka, K., Koshikawa, F. Dorsolateral prefrontal cortex sensing analgesia. Biophys. Physicobiol. 19, e190014 (2022). https://doi.org/10.2142/biophysico.bppb-v19.0014 10.2142/biophysico.bppb-v19.0014 PMC9173858 35797407

[17] Lee, C. H., Sugiyama, T., Kataoka, A., Kudo, A., Fujino, F., Chen, Y. W., et al. Analysis for distinctive activation patterns of pain and itchy in the human brain cortex measured using near infrared spectroscopy (NIRS). PLoS One 8, e75360 (2013). https://doi.org/10.1371/journal.pone.0075360 10.1371/journal.pone.0075360 PMC3789686 24098378

[18] Hu, C., Kato, Y., Luo, Z. An fNIRS study of taste cortical areas in human brain: Sweetness and sourness. in Proceedings of the 2013 IEEE/SICE International Symposium on System Integration, Kobe, Japan, 15–16 December 2013.https://doi.org/10.1109/SII.2013.6776655

[19] Herwig, U., Satrapi, P., Schönfeldt-Lecuona, C. Using the international 10-20 EEG system for positioning of transcranial magnetic stimulation. Brain Topogr. 16, 95–99 (2003). https://doi.org/10.1023/b:brat.0000006333.93597.9d 10.1023/b:brat.0000006333.93597.9d 14977202

[20] Talairach, J., Tournoux, P. Co-planar Stereotaxic Atlas of The Human Brain: 3-D Proportional System: An Approach to Cerebral Imaging (Georg Thieme Verlag, Stuttgart, Germany, 1988).

[21] Jensen, K. B., Berna, C., Loggia, M. L., Wasan, A. D., Edwards, R. R., Gollub, R. L. The use of functional neuroimaging to evaluate psychological and other non-pharmacological treatments for clinical pain. Neurosci. Lett. 520, 156–164 (2012). https://doi.org/10.1016/j.neulet.2012.03.010 10.1016/j.neulet.2012.03.010 PMC3810294 22445888

[22] Du, R., Lao, M., Cheng, M., Wang, Z., Zheng, Z., Tang, P., et al. Working memory load-dependent cortical mechanism of distraction analgesia in healthy individuals: An fNIRS study. J. Pain Res. 18, 5599–5615 (2025). https://doi.org/10.2147/JPR.S546795 10.2147/JPR.S546795 PMC12572738 41180365

[23] Sandri, A., Cecchini, M. P., Riello, M., Zanini, A., Nocini, R., Fiorio, M., et al. Pain, smell, and taste in adults: A narrative review of multisensory perception and interaction. Pain Ther. 10, 245–268 (2021). https://doi.org/10.1007/s40122-021-00247-y 10.1007/s40122-021-00247-y PMC8119564 33635507

